# ATP1B3 may promote glioma proliferation and migration through MAPK/NF-KB signaling pathway

**DOI:** 10.3389/fonc.2025.1537687

**Published:** 2025-02-11

**Authors:** Qikang Yan, Quan Sun, Yan Feng, Qingyun Hu, Jinling Zhu

**Affiliations:** ^1^ School of Basic Medicine, Jiamusi University, Jiamusi, China; ^2^ Qilufoshan Community Hospital, People's Hospital of Lixia District, Jinan, Shandong, China

**Keywords:** glioma, ATP1B3, proliferation, migration, MAPK signaling pathway, NF-κB signaling pathways

## Abstract

**Objective:**

To investigate the function of ATPase Na+/K+ Transporting Subunit Beta 3 (ATP1B3) in gliomas and the molecular mechanisms associated with them in order to identify a novel target and approach for glioma clinical diagnosis and treatment.

**Methods:**

The Cancer Genome Atlas (TCGA), a public tumor database, and the Chinese Glioma Genome Atlas (CGGA) were used to evaluate the differential expression of ATP1B3 in glioma cells of various grades. Its connection to patient survival and prognosis; The siRNA interference approach instantly reduced the amount of ATP1B3 expression in the glioma cell lines U87MG and U251MG. The knockdown efficiency was assessed by Western Blotting (WB) and RT-qPCR. Following ATP1B3 knockdown, the ability of glioma cells to proliferate, migrate, and invade was identified using the Transwell assay and CCK-8. The proteins that might interact with ATP1B3 were filtered out using the TCGA database and literature analysis. The WB assay was used to determine the expression level of Protein Phosphatase 1 Catalytic Subunit Alpha (PPP1CA) following ATP1B3 deletion, immunoprecipitation was used to determine the direct influence of the two proteins, and immunofluorescence was used to analyze the distribution of ATP1B3 and PPP1CA proteins in glioma cells. Cyclin D1 and vascular endothelial growth factor A(VEGFA) expression alterations following ATP1B3 deletion were identified using the WB assay. Following ATP1B3 knockdown, the WB assay was used to determine the expression levels of p-Raf1, p-MEK 1/2, p-ERK 1/2, p-IκBα, and p-P65 in the MAPK and NF-κB signaling pathway.

**Results:**

Database analysis revealed a negative correlation between the patients’ prognosis and the expression level of ATP1B3, and a positive correlation with the malignant degree of the glioma. The mRNA and protein expression levels of ATP1B3 were significantly decreased after knockout, and the proliferation, migration and invasion ability of cells in knockout group were significantly lower than those in control group, with statistical difference. The immunoprecipitation results were negative, and the knockdown group’s PPP1CA expression was lower than the control group’s. Following ATP1B3 knockdown, Cyclin D1 and VEGFA protein expression levels dropped, and the effects were statistically significant. There was a statistically significant drop in the expression levels of p-Raf1, p-MEK 1/2, p-ERK 1/2, p-IκBα, and p-P65 following ATP1B3 deletion.

**Conclusion:**

In gliomas, ATP1B3 is highly expressed. Glioma cell motility, invasion, and proliferation all decline when ATP1B3 expression is lowered. The downstream protein PPP1CA is indirectly regulated by ATP1B3. By controlling the MAPK and NF-κB signaling pathways, ATP1B3 may have a role in the invasion, migration, and proliferation of glioma cells. As a result, the ATP1B3 gene might be a biological target for treatment and a possible neurotumor diagnostic.

## 1 Introduction

Gliomas, the most prevalent and deadly kind of brain tumor, originate from glial cells of the central nervous system. They develop quickly and are very aggressive, and patients have a dismal prognosis (1). The fifth edition of the WHO-CNS5 (Central Nervous System Tumor Classification) will be released in 2021. In addition to integrating a stratified diagnosis by combining histopathological findings and immunohistochemical techniques, WHO-CNS5 integrates features including gene, molecular, and signaling pathway alterations into the categorization system (2–4). Gliomas of grades 1, 2, 3, and 4 are treated differently, which results in a markedly variable prognosis and survival rate for patients. Surgery is the main treatment for Lower Grade Glioma (LGG) (grades 1 and 2). Regardless of the extent of surgical resection, follow-up adjuvant therapy, such as chemotherapy and radiation, is necessary for High Grade Glioma (HGG) (grades 3 and 4). Pilocytic astrocytoma, pleomorphic xanthoastrocytoma, and ependymal giant cell astrocytoma are examples of low-grade gliomas (5). Anaplastic astrocytoma, anaplastic ependymoma, anaplastic oligodendroglioma, and anaplastic oligastroglioma are examples of high-grade gliomas (6). Glioblastoma multiforme (GBM) is classified as WHO Grade 4 and is extremely malignant. Uncontrolled cell proliferation is one of the most evident characteristics of GBM (7). The complicated processes that drive proliferation include a variety of external inputs, membrane receptor systems, intracellular signaling pathway networks, and alterations in certain genes (8). K-ATP/NaAse, also referred to as the NA-K pump, is an embedded protein in the plasma membrane of all animal cells that hydrolyzes ATP to move sodium and potassium ions both within and outside of the cell while preserving the cell’s electrochemical gradient and ion concentration balance (9). The catalytic α (100–112 kDa), regulatory β (45–55 kDa), and γ (6.5–10 kDa) subunits make up Na/K-ATPase (10). The beta subunit has been shown to have three different isotypes: β1 (ATP1B1 gene), β2 (ATP1B2 gene), and β3 (ATP1B3 gene) (11). The kidney, brain, and heart are the primary tissues where the three beta subunits are distributed in cells (10). In mammalian epithelial cells, polarity is formed by the Na/K-ATPase β subunit, and tight junction and polarity development are weakened when the β1 subunit is absent (11). T Located in the cell junction region, the glycosylation portion of the n-junction of the extracellular component of the β1 subunit is linked to ERK1/2 activation (12). According to some research, the Na/K-ATpase β3 subunit not only contributes to the functional activity of Na/K-ATPase but also functions as a receptor molecule that controls immune cells, prevents T cell proliferation, and aids in tumor tissue angiogenesis (13). Low expression of ATP1B3 decreased the proliferation of human venous endothelial cells and enhanced cell death, according to Mesri et al. (14)who employed the experimental approach of mass spectrometry to look for therapeutic targets that may be exploited to inhibit tumor angiogenesis. Three β subunits of Na/K-ATPase were found to be highly expressed in gliomas, according to Deborah Rotoli et al. (15). β1 was expressed in all cells except astrocytes, and β2 and β3 were expressed in a variety of cells. However, as the disease severity increased, β3 expression was abnormally enhanced and β2 expression was weakened. Research has shown that when ATP1B3 expression is knocked down, the proliferation, migration, and invasion capacity of gastric cancer cells are significantly decreased, and the mRNA and protein levels of ATP1B3 are elevated in gastric cancer cell lines when compared to normal gastric epithelial cell lines (16). However, there is a dearth of domestic and international research on the mechanism of action of ATP1B3 and its role in gliomas. Investigating ATP1B3 expression and mechanism in glioma cells was the goal of this work.

## 2 Materials and methods

### 2.1 Database

The expression of ATP1B3 in gliomas of various grades (grades 2, 3, and 4) was examined online using the CGGA database website. Using the TCGA database’s transcriptome data for the samples: Based on the median expression level of ATP1B3, the sample data of glioma patients were separated into two groups: the group with high ATP1B3 expression and the group with low ATP1B3 expression. Overall Survival (OS), Progress Free Surviva (PFS), and Receiver Operating Characteristic Curve (ROC) survival analysis were among the R packages “survival” and “survminer” that were utilized to examine the correlation between the ATP1B3 gene’s expression level and the survival rate of glioma patients. We compared and examined the relationship between the expression level of ATP1B3 and the clinical features of cancer patients, such as grade, age, and gender. ATP1B3 was used as a key word in the String database to check for the interacting proteins.

### 2.2 Cell culture

After taking the U87MG, U251MG, and HMC3 cells out of the liquid nitrogen tank, place them in water that is 37 ℃. After melting, the cells were extracted from the frozen tube and centrifuged for five minutes at 900 RPM in a 10 mL tube that contained eight milliliters of media. After adding 1 mL of medium for re-suspension and discarding the supernatant, the cells were placed in the culture bottle, 4 mL of medium was added, and the microscope was used to observe the cells. The cells were cultivated in a medium that contained 1% glutamine solution, 1% cyanomycin solution, and 10% fetal bovine serum. HMC3 and U87MG were placed in MEM medium, while U251MG was placed in DMEM high-sugar medium.

### 2.3 Quantitative real-time PCR

The RNAeasyTM animal RNA extraction kit (centrifugal column) was used to extract total RNA. The BeyoRT™ II cDNA First Strand Synthesis Kit (RNase H-) was used to amp up cDNA from RNA sources. Following the manufacturer’s instructions, PCR amplification was carried out in the Applied Biosystem 7300 Real-Time PCR System (Applied Biosystems, Foster, CA, USA) using the ROX Reference Dye Kit (Takara). Stage 2: PCR reaction, Reps: 40, 95°C for 5 seconds, 60°C for 31 seconds, and 40°C for storage; Stage 1: predenaturation, Reps: 1, 95°C for 30 seconds. The 2^-ΔΔCT^ was used to standardize the relative gene expression.

### 2.4 Western blot

Total proteins extracted from HMC3, U87MG and U251MG cells with Western and IP cell lysate (Beyotime) were quantified using Bio sharp assay kit. The 40 μg protein was transferred using a PVDF membrane (Merck Millipore) and added to the swim lane of 10% SDS-PAGE gel. Primary antibody including ATP1B3 (1:000, Proteintech), p-MEK1/2 (1:1000, Proteintech), MEK1/2 (1 × 1000, Abcam), NF-κB/p65 (1:1000, Proteintech), p-NF-κB/p65 (1:1000, Affinity), ERK1/2 (1:1000, Proteintech), p-ERK1/2 (1:1000, Proteintech), Anti-Rabbit IgG (1:2000; Beyotime) and anti-mouse IgG (1:2000; Beyotime) for incubation. An enhanced ECL chemiluminescence substrate kit [Yeasen Biotechnology (Shanghai)] was used and quantification was performed by ChemiDoc Imaging system (Bio-Rad Laboratories, Inc).

### 2.5 Cell transfection

The cultivated cells were separated into five groups: blank control group (Ctrl), negative control group (si-Ctrl), si-ATP1B3-336 group, si-ATP1B3-530 group, si-ATP1B3-976 group, and SI-ATP1B3-336 group. Gimar Genomics designed the interference RNA sequence. After the transfection system is developed and the cell density in the 6-well plate reaches 30% to 50%, transfection can begin. 100 pmol of siRNA (negative control group was added to negative control siRNA, si-ATP1B3-HOM-336 group was added to ATP1B3-HOM-336 group, si-ATP1B3-530 group was added to ATP1B3-HOM-530 group, and si-ATP1B3-976 group was added to ATP1B3-HOM-976 group), 125 μL of OPTI-MEM, and 4 μL of Lipo-8000 were added. The mixture was thoroughly mixed and allowed to stand at room temperature for 20 minutes. A 6-well plate containing U87MG and U251MG cells was dropped with the transfection agent, shook gently, and then cultivated in a cell incubator for 24 hours for RT-qPCR detection and transfected cells for 48 hours for WB detection. High knockdown effectiveness siRNA was selected for further research.

### 2.6 Cell Counting Kit-8 assay

In a 96-well plate, cells from the si-ATP1B3-336 and control groups were planted (3×10^3 cells/well), with five replicates for each group. Following the reagent instructions, 10 μL of CCK-8 solution was applied to each well, and the wells were then incubated for an hour. A microplate reader was used to measure the optical density (OD) at 450 nm. Every well’s values were constantly recorded at 0, 24, 48, and 72 hours. The richer the hue and the greater the OD value, the faster the cells multiply.

### 2.7 Transwell assay

The upper chamber was filled with a suspension of U87MG and U251MG cells in 200μL of fetal bovine serum medium. The lower chamber was filled with roughly 600 µL of medium that contained 10% fetal bovine serum. The basal cells were fixed with 4% formaldehyde and stained with Giemsa following a 24-hour incubation period. Lastly, five randomly chosen fields were observed in order to assess the cells’ capacity for invasion.

### 2.8 Co-immunoprecipitation

After lysing U87MG and U251MG cells in media, the supernatant was treated for 1.5 hours at 4°C with 2μg of ATP1B3 and PPP1CA antibodies. Following another 1.5 hours of addition of protein A/G-agar-agar beads (Biyun Tian), the buffer was added to the PBS-rinsed pellets, which were then incubated at 95°C for 5 minutes before being identified by Western blotting.

### 2.9 Statistical analysis

Prism 9 (GraphPad Software, USA) was used to analyze the data, which were presented as mean ± SD and subjected to either an ANOVA or the Student t test. The threshold for statistical significance was set at P <0.05.

## 3 Result

### 3.1 ATP1B3 is highly expressed in glioma

**Figure 1:**
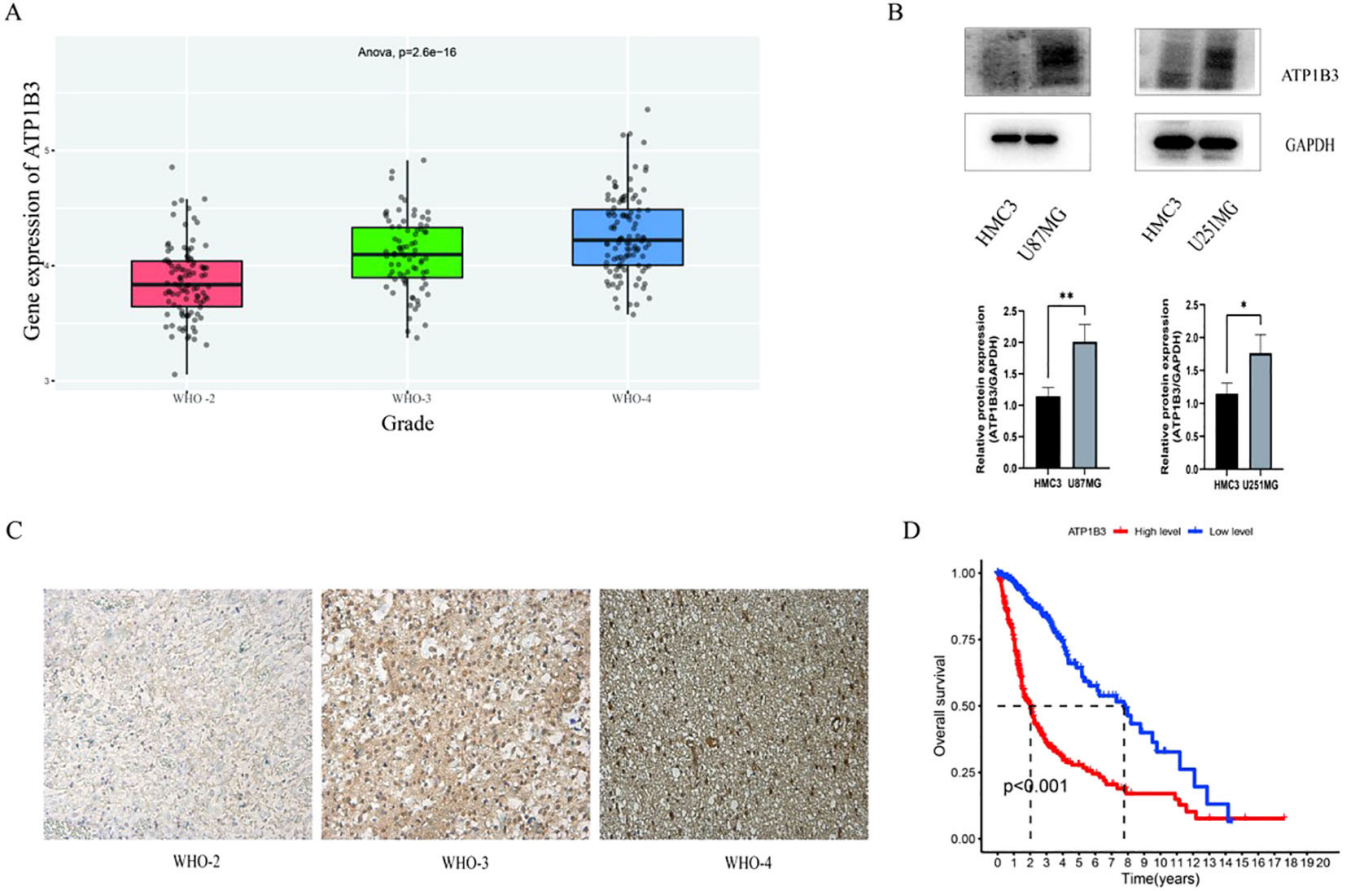
Expression of AT1PB3 varies by glioma grade. **(A)** Analysis of the CGGA database ATP1B3 expression is positively correlated with glioma grade (P<0.001). **(B)** U87MG and U251MG had considerably greater levels of ATP1B3 expression than HMC3 **P<0.01 and *P<0.05, respectively. **(C)** Analysis via immunohistochemistry. On a scale of 50 μm, grade 2 is yellowish +, grade 3 is yellowish brown ++, and grade 4 is brown +++. **(D)** Patients with high ATP1B3 expression had a considerably lower productivity than those with low expression, with a P-value of less than 0.001.

The CGGA website was used to examine the expression level of ATP1B3 in gliomas of various grades. The results indicated that the expression level of ATP1B3 rose considerably with the grade of glioma (grade 2, grade 3, grade 4), with a P-value of less than 0.001 (**Figure 1A**). Glioma cell lines U87MG and U251MG have significantly greater levels of ATP1B3 expression than the normal glioma cell line HMC3, as illustrated in **Figure 1B**. *P<0.05, **P<0.01. The findings of database analysis were confirmed by immunohistochemical results, which revealed that grade 2, 3, and 4 glioma tissue sections displayed pale yellow, yellowish brown, and brown, respectively. The darker the color, the higher the expression level. There are four distinct staining intensity scores: - (no staining), + (weak staining), ++ (medium staining), and +++ (high staining) (**Figure 1C**). 704 glioma samples were taken from TCGA data in order to better understand how ATP1B3 expression affects glioma patient survival. The independence test was then performed for the two groups with high and low ATP1B3 expression, and R software was used to analyze the patients’ survival duration. The impact of ATP1B3 expression level on the overall survival rate of glioma patients was calculated using the R language package, and a survival curve was created. Compared to the group with high ATP1B3 expression, the group with low ATP1B3 expression had a considerably greater survival rate (P<0.001) (**Figure 1D**).

### 3.2 The knockdown efficiency of interfering RNA si-ATP1B3-336 group was the most significant

**Figure 2:**
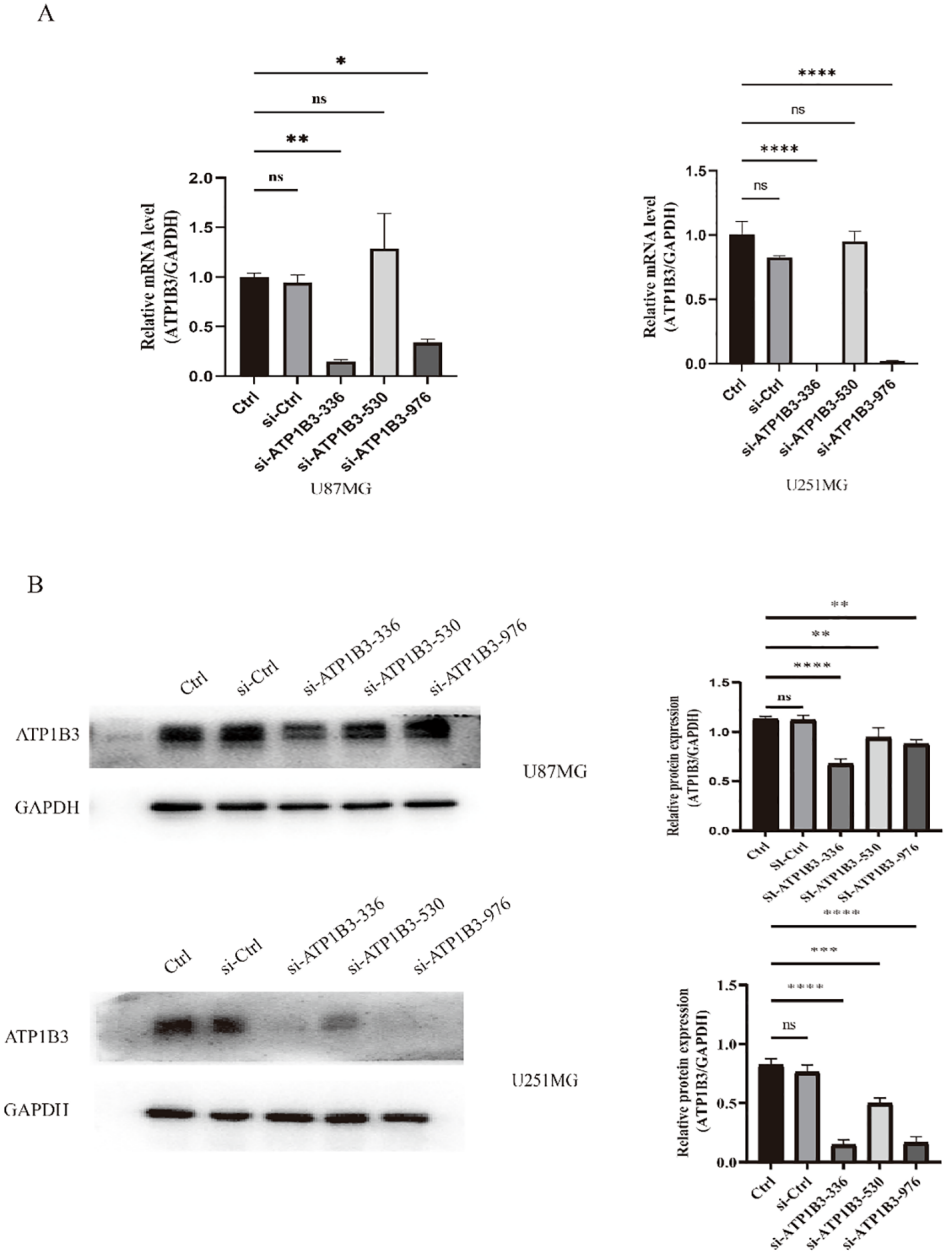
Verification through experimentation of ATP1B3 expression knockdown in glioma cell lines. **(A)** The si-ATP1B3-336 group had a considerably lower amount of ATP1B3 mRNA, according to fluorescence quantitative PCR data (ns P>0.05, no significant difference, *P<0.05, **P<0.01, ****P<0.0001). **(B)** The si-ATP1B3-336 group had considerably lower amounts of ATP1B3 protein, according to the results of the western blot (ns P>0.05, no significant difference, **P<0.01, ***P<0.001, ****P<0.0001).

To lower the expression of ATP1B3, si-ATP1B3-336, si-ATP1B3-530, and si-ATP1B3-976 were transfected into U87-MG and U251-MG cell lines for knockdown, respectively. Testing was done using RT-qPCR and WB, and the outcomes are displayed in **Figure 2**. The si-ATP1B3-336 group exhibited the most pronounced knockdown effect, according to the RT-qPCR data, and the transcription level of ATP1B3 was dramatically reduced (**P<0.01, ****P<0.0001). The si-ATP1B3-336 group exhibited the most pronounced knockdown effect, according to the results of the WB test, and the expression level of the ATP1B3 protein was dramatically reduced (****P< 0.0001, ***P< 0.001).

### 3.3 Interferes with ATP1B3 to inhibit GBM cell proliferation, migration and invasion

**Figure 3:**
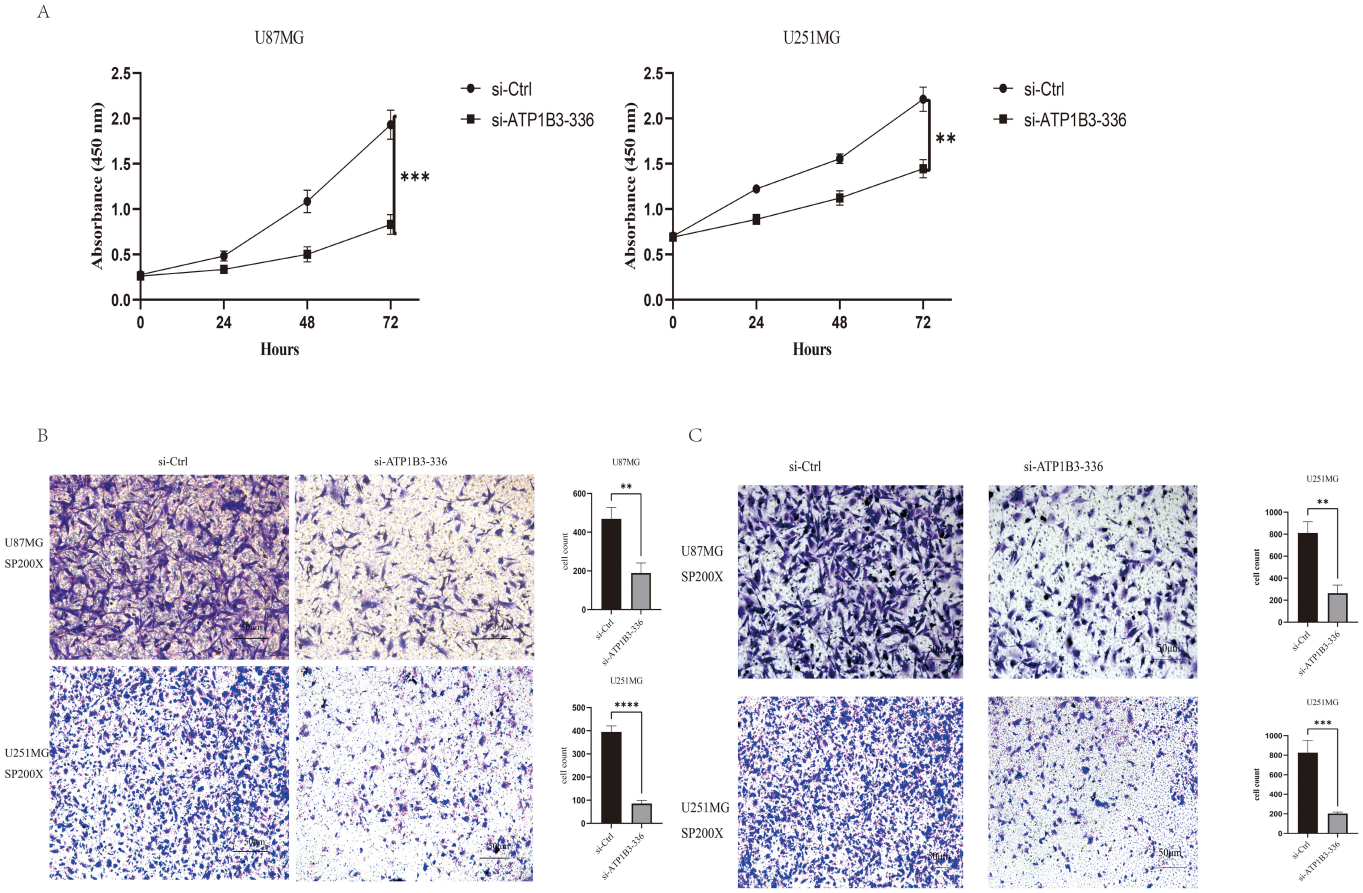
The phenotypic alterations of glioma cells are impacted by the downregulation of ATP1B3. **(A)** Following ATP1B3 knockdown in U87MG and U251MG cells, the control group’s absorbance was considerably greater than the knockdown group’s, indicating a significant decrease in the proliferative ability of the knockdown group’s cells (***P<0.001, **P<0.01). **(B)** The knockdown group had significantly fewer U87MG and U251MG cells than the control group, **P<0.01, ****P<0.0001.**(C)** Transwell analysis of each group’s capacity for cell invasion revealed that the knockdown group had significantly less U87MG and U251MG cells than the control group, **P<0.01, ***P<0.001.

In the CCK-8 cell proliferation assay, U87MG and U251MG cell proliferation was dramatically reduced (P<0.001) by knockdown ATP1B3 expression in comparison to the si-NC group (**Figure 3A**). **Figures 3B, C** demonstrate how ATP1B3 knockdown dramatically reduced U87MG and U251MG cell migration and invasion.

### 3.4 Database screening of proteins significantly associated with ATP1B3

**Figure 4:**
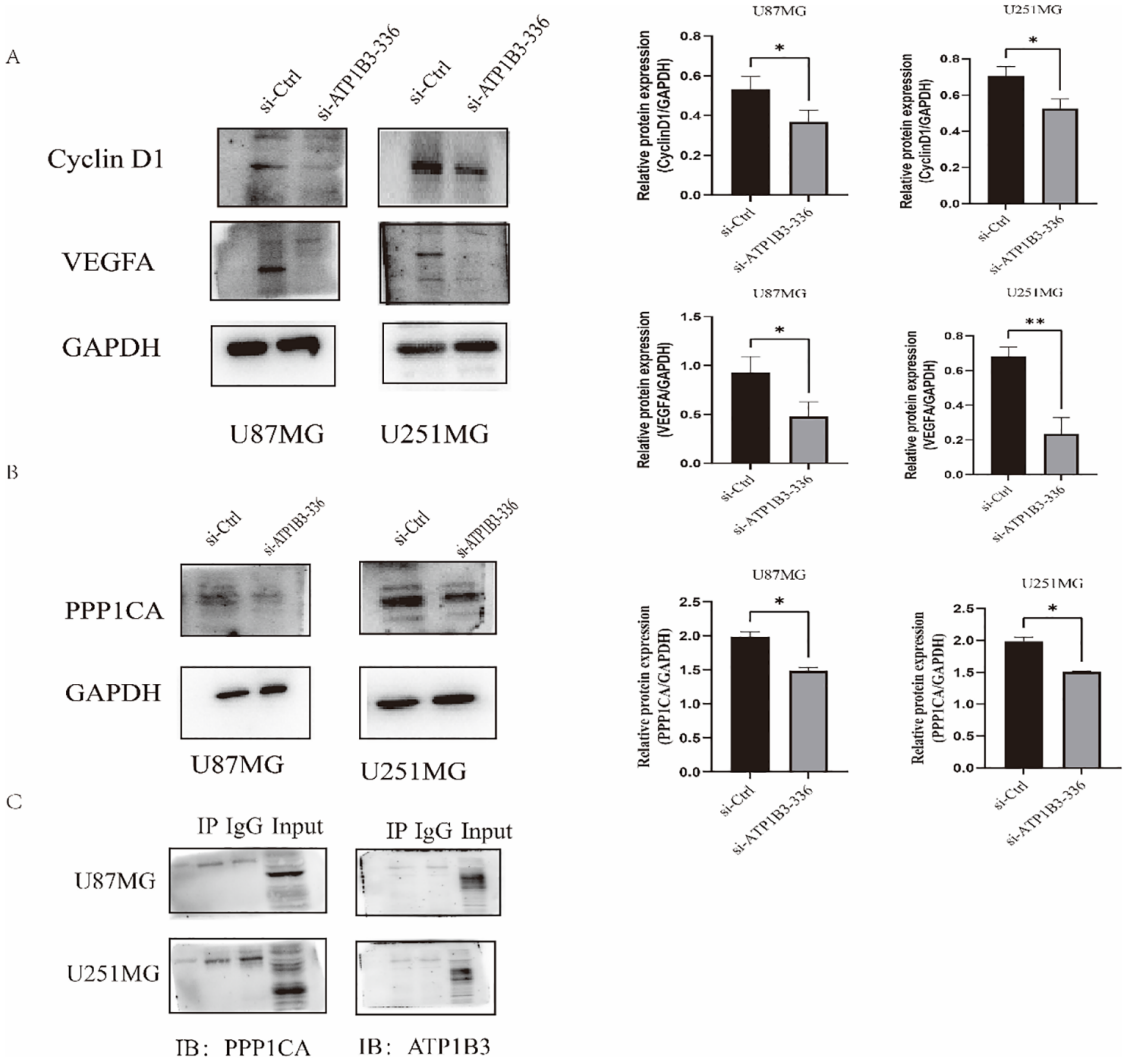
**(A)** Cells in the knockdown group had considerably lower levels of Cyclin D1 and VEGFA protein expression (*P<0.05, **P<0.01). **(B)** The knockdown group’s PPP1CA protein expression level in U87MG and U251MG was considerably lower than the control group’s, *P<0.05. **(C)** Immunoprecipitation experiments showed no interaction between ATP1B3 and PPP1CA, and the results were negative, PPP1CA interacts indirectly with ATP1B3.

We employed the WB assay to identify Cyclin D1 and VEGFA proteins in order to investigate the impact of ATP1B3 expression on the cell cycle and angiogenesis. The outcomes are displayed in **Figure 4**. In U87MG and U251MG cells, the ATP1B3 knockout group’s Cyclin D1 protein expression was significantly lower than that of the control group (P<0.05), while the knockout group’s VEGFA protein expression was significantly lower than that of the control group (P<0.05), P<0.01. ATP1B3 might be connected to the angiogenesis of gliomas. After downloading and analyzing glioma transcription data from the TCGA database, 202 distinct proteins were tested for correlation coefficients more than 0.65 and P<0.001. The glioma’s malignant growth was aided by the correlation coefficient of 0.67 with ATP1B3. The CGGA database was used to examine the expression level of PPP1CA in gliomas of various grades online. The findings demonstrated a substantial rise in PPP1CA expression level when glioma grade (grade 2, 3, and 4) increased (P<0.001). The group with low PPP1CA expression had a considerably greater survival rate than the group with high PPP1CA expression (P<0.001). Using immunofluorescence, the locations of ATP1B3 and PPP1CA in glioma cells were determined. The connection between the two proteins was made feasible by the distribution of PPP1CA protein throughout the cytoplasm, nucleus, and cell membrane, whereas ATP1B3 protein was primarily found in the cell membrane. The protein expression of PPP1CA was considerably (P<0.05) reduced following ATP1B3 knockdown. When ATP1B3 and PPP1CA were examined using a co-immunoprecipitation technique, the results indicated that there was no direct contact between the two.

### 3.5 ATP1B3 affects MAPK and NF-κB signaling pathways

**Figure 5:**
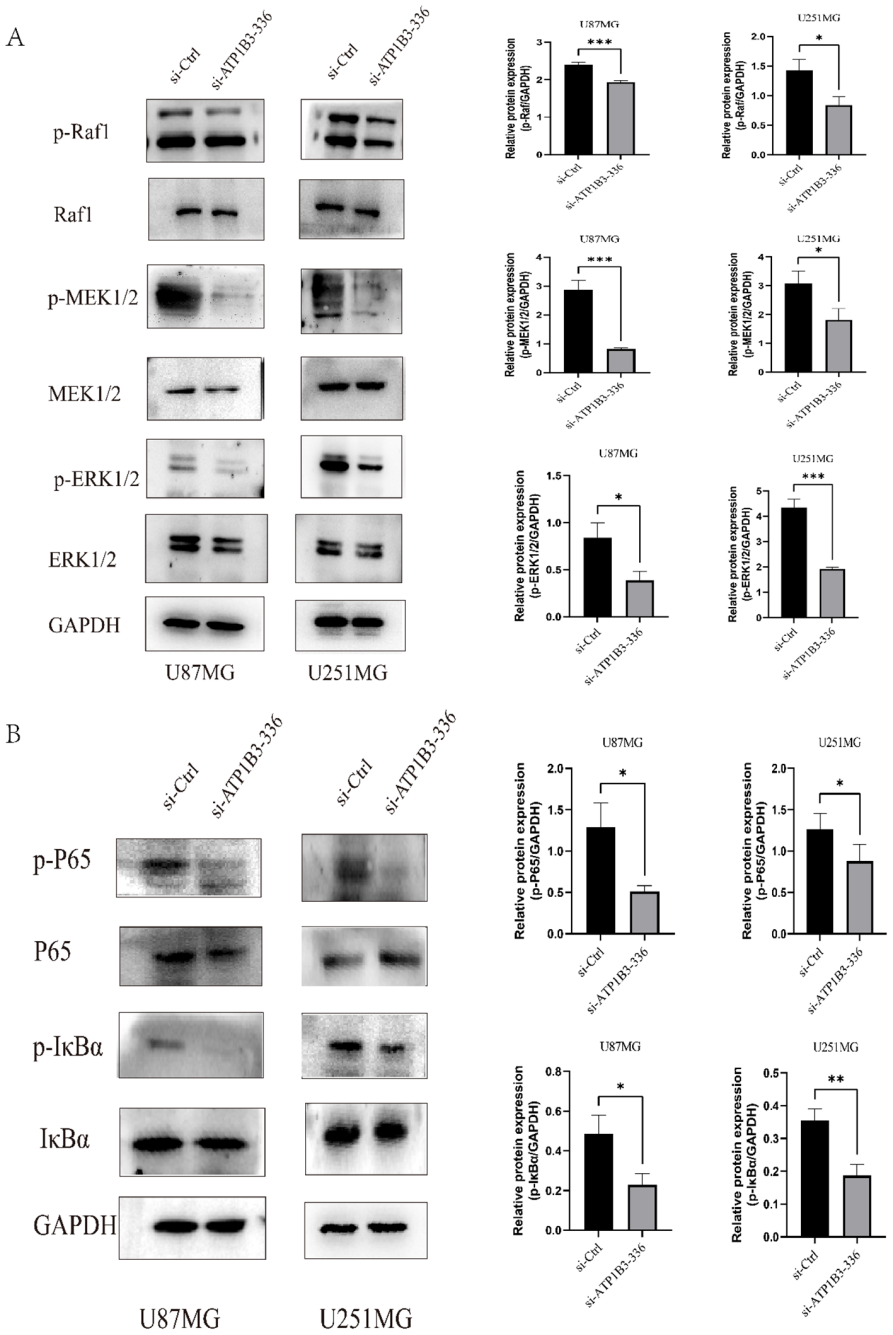
The MAPK and NF-κB signaling pathways are impacted by ATP1B3 expression. **(A)** The knockdown group cells had considerably lower levels of p-Raf1, p-MEK 1/2, and p-ERK 1/2 protein expression than the control group cells (*P<0.05, ***P<0.001). Raf1, MEK 1/2, and ERK 1/2 protein expression remained essentially unaltered. **(B)** While the expression levels of IκBα and P65 proteins were essentially intact, the expression levels of p-IκBα and p-P65 proteins decreased in U87MG and U251MG cells, *P<0.05, **P<0.01.

The phosphorylated Raf1-MEK 1/2-ERK 1/2 alterations in the MAPK signaling pathway, as seen in **Figure 5A**, were confirmed by the WB test to result in a decrease in the levels of p-Raf1, p-MEK 1/2, and p-ERK 1/2 protein expression. Raf1, MEK 1/2, and ERK 1/2 expression levels did not alter. Therefore, by altering the MAPK signaling pathway, ATP1B3 may have an impact on the malignant development of gliomas. **Figure 5B** shows the modifications of p-IκBα and p-P65 in the NF-κB signaling pathway after the ATP1B3 changes were confirmed by WB assay. U87MG and U251MG cells showed lower levels of p-IκBα and p-P65 protein expression following the knockdown of ATP1B3 expression. P65 and IκBα expression levels did not change.

## 4 Discussion

For academics both domestically and internationally, the fundamental study of gliomas and the investigation of their therapy have long been a contentious and challenging issue. Finding and studying the molecular and functional genes linked to the malignant evolution of gliomas, as well as gaining a thorough understanding of the possible molecular regulatory mechanisms of glioma progression, has long been the main approach for glioma prevention and treatment. As early as 2013, ATP1B3 and other angiogenesis targets identified by endothelial cells in human cancer tissues were discovered using proteomic analysis (14). Though there are currently few precise molecular mechanistic investigations on its involvement, it has been confirmed thus far that ATP1B3 plays a role in a number of malignancies and has the potential to become a new target for clinical therapy of cancer.

In this work, we investigated in detail how ATP1B3 promotes the migration, invasion, and proliferation of glioma cells. By influencing the MAPK/NF-κB and PPP1CA signaling pathways, ATP1B3 may control the malignant development of gliomas.

### 4.1 ATP1B3 is expressed in glioma and correlated with clinical features of patients

Few studies have examined the impact of ATP1B3 in gliomas, despite numerous studies demonstrating its pro-cancer role in some malignancies. As a result, this study’s online examination of gliomas of various degrees (grades 2, 3, and 4) in the CGGA database revealed that, with a statistical difference (P<0.05), the expression level of ATP1B3 also increased significantly as glioma grade increased (**Figure 1**). Concurrently, immunohistochemistry confirmation was performed on the glioma tissue sections that were collected, and the findings likewise verified that ATP1B3 expression increases with the malignant degree of the glioma. After that, the WB experiment was used to determine the level of ATP1B3 expression in the U87MG, U251MG, and HMC3 cell lines (**Figure 1**). Glioma cells had significantly more ATP1B3 than the normal group (P<0.05). Analysis of the TCGA database revealed a correlation between the clinical features of glioma patients and ATP1B3 expression. The clinical information of glioma patients that was retrieved from the TCGA database was used to create the survival curve. **Figure 3** illustrates how ATP1B3 had a particular stimulating influence on the malignant development of gliomas and how high expression of the protein was positively connected with patients’ bad prognoses. This is consistent with the results of current studies (17–19), which preliminarily confirms that ATP1B3 is an oncogene and is associated with poor prognosis. ATP1B3 is expected to be a new potential glioma molecular marker.

### 4.2 High expression of ATP1B3 promotes proliferation, migration and invasion of glioma cells

Tumor cells multiply endlessly in the primary site during the early stages of multi-stage tumor progression, and it frequently takes years for the formation of visible primary tumor lesions (20). When these growths and tumors reach a particular size across the body, they will impact the organ’s function (21), leading to varying degrees of clinical symptoms. About 90% of patients eventually experience life-threatening metastases that expand outside the initial location, whereas only 10% of patients die from primary tumors, despite the fact that they are extremely deadly (22, 23). The most damaging stage of tumor development is metastasis, and once tumor cells proliferate and spread to other organs, the body will suffer severe consequences (24, 25). The traits of the tumor cells themselves as well as the intricate biochemical and biological alterations in the surrounding matrix mostly determine how aggressive the tumor is (26). Nonetheless, we still don’t fully understand the mechanisms underlying tumor invasion and metastasis, and the primary unanswered questions in the study of tumor pathogenesis are those related to invasion and metastasis that occur in the latter stages of tumor evolution.

In this study, we employed RNA interference technology to inhibit ATP1B3 expression in glioma cells. Following this, we used the CCK-8 experiment, plate cloning formation experiment, and Transwell laboratory experiment to observe changes in the proliferation, migration, and invasion capacity of U87MG and U251MG. **Figure 3** displays the absorbance values of the control group and the knockout group at 0 hours, 24 hours, 48 hours, and 72 hours, respectively. The results showed a statistically significant difference (P<0.01) in the knockout group’s ability to proliferate cells compared to the control group. Transwell migration and invasion assays revealed that U87MG and U251MG’s migration and invasion capacity significantly decreased (P<0.05) following the knockdown of ATP1B3 expression, as illustrated in **Figure 3**. Thus, it has been established that ATP1B3 expression is linked to the growth, migration, and invasion of glioma cells; however, additional *in vivo* research is required to confirm that ATP1B3 contributes to the development of cancer in gliomas.

## Data Availability

Publicly available datasets were analyzed in this study. This data can be found here: https://portal.gdc.cancer.gov/car, and https://www.cgga.org.cn/analyse/RNA-data.jsp, distribution gene "ATP1B3".
